# Surveillance in the lab? How datafication is changing the research landscape

**DOI:** 10.1038/s44319-024-00153-2

**Published:** 2024-05-10

**Authors:** Stephan Guttinger

**Affiliations:** Department of Social and Political Sciences, Philosophy and Anthropology (SPSPA), Egenis Centre for the Study of the Life Sciences, University of Exeter, Exeter, UK

**Keywords:** Economics, Law & Politics, History & Philosophy of Science, Science Policy & Publishing

## Abstract

Scientific research is increasingly becoming datafied through the use of electronic lab notebooks and smart instruments. This has significant implications for surveillance at work and research itself.

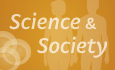

Scientific research is increasingly “datafied” through the proliferation of smart instruments, Electronic Laboratory Notebooks (ELNs) and cloud-based applications and services for storing or analysing data. This offers new opportunities for optimising the efficiency and reproducibility of the research process, but it also raises important ethical and methodological questions for individual researchers and research institutions. The “datafication” of science has not yet received much attention from scientists, philosophers or other scholars who study the sciences. We therefore lack the conceptual and empirical foundations for a critical assessment of this process and its potential ramifications. Here I will describe what datafication means in the context of science; highlight two of the potential issues it raises; and present a framework that could support practitioners when they navigate the increasingly datafied research environment.

## What is the “datafication” of science?

The term “datafication” was coined to capture a phenomenon that goes beyond mere digitalisation: datafication is not just about transforming analogue data into a digital format. It is about what is captured as data in the first place (Cukier and Mayer-Schoenberger, 2013). More specifically, it is about how more and more aspects of our daily lives are turned into digital traces: from our interactions with friends and family, to our likes and dislikes, the professional networks we form, or even just the act of moving about. These data can be collected, stored and analysed in order to predict and manipulate human behaviour (Zuboff, 2015).

Although science has been at the forefront of digitalisation over the past few decades, it has for long resisted datafication in the sense of Cukier and Mayer-Schoenberger. Most research data—be they generated through experiments, measurements, observations or surveys—are now stored and processed in a digital format. In fact, digitalisation has become a *sine qua non*: new technologies, such as high-resolution microscopy or next-generation sequencing produce enormous amounts of data that require further processing and analysis by high-powered computational tools. The wider sharing of these data also necessitates digital formats and cloud-based services—Open Science would be an even more difficult exercise if it were based on the storage and shipping of paper files.

“Although science has been at the forefront of digitalisation over the past few decades, it has for long resisted datafication…”

But this digitalisation of research outputs does not necessarily amount to a datafication of science itself. Indeed, science has long resisted being datafied. For most of its history, scientists used pen and paper notebooks to record aspects of their experimental practice, such as the day on which an experiment was performed, or the reagents and experimental conditions they used. They still organise the use of shared instruments via paper forms attached to the instrument, or via simple booking systems on local networks. They usually manage the stock and the purchase of reagents on an ad hoc basis with Excel spreadsheets.

In addition, various aspects of research practice are not recorded at all. Scientists usually do not keep a record of all the people they shared their experimental results with. They do not keep a record of when they open or close their laboratory notebook, or which entry they looked at. They do not record how much time they spent to think about and plan an experiment, or who they discussed their ideas with.

However, this somewhat archaic underbelly of the research process is being re-imagined and re-designed. Scientific research is becoming rapidly datafied through the proliferation of smart instruments and cloud-based applications. Experiments are increasingly planned, recorded and shared using cloud-native ELNs (Kwok, 2018). The daily flow of reagents and the use of automated instruments are managed and monitored via cloud-based Laboratory Information Management Systems (LIMSs) (Perkel, 2015). Experiments are performed on “smart” instruments, which can feed results—and activity data—directly into an ELN or LIMS. In some cases, whole experiments are performed off-site in “cloud laboratories” (Arnold, 2022).

“Scientific research is becoming rapidly datafied through the proliferation of smart instruments and cloud-based applications.”

This move to what I will call “cloud-enabled research” (CER) offers new opportunities for optimising the efficiency and reliability of the research process. Similar to the idea of “Open Science” (Mirowski, 2018), the automation of specific processes is now presented as a panacea for the problems that ail contemporary science. A good example is the problem of reproducibility: almost every provider of ELNs, smart instruments or cloud laboratories sells their tool or workflow with the promise that it will vastly improve the reproducibility of the experimental work that is done with or through it.

But whilst it will have its benefits, the process of datafication also raises important methodological and ethical questions. Who has access to the data that is collected and why? How do the new data flows change everyday research practice? Are scientists being subjected to “dataveillance”, and if so, what does this mean for their privacy? To ensure that the scientific community embarks on a critically informed journey towards a datafied research ecosystem, these questions need to be addressed.

## Surveillance and privacy in a datafied research environment

A key issue of datafication is surveillance and the impact this might have on privacy. Smart tools and ELNs turn a researcher’s every move in the workplace into data. ELNs not only log when a researcher opens their personal notebook. They might also log whom a researcher talks to (chat function); who they share files with and when they do so (integrated data sharing tools); which instruments they use for which purpose (instrument integration); what materials were used for what experiment (inventory management functions); and how data was processed (integrated data analysis tools). This in-depth tracking of activities certainly has benefits for the traceability and reproducibility of workflows. But it also raises important questions about privacy and legitimate access to data.

The question of privacy in the workplace has become an increasingly important topic since the 1990s, when the adoption of email and the use of the Internet opened new avenues for employee surveillance (Miller and Weckert, 2000). But the level of surveillance that comes with CER goes beyond the monitoring of emails and browsing activity, as it allows employers to collect much more detailed information about a researcher’s daily practice.

Such “dataveillance” can impact autonomy and trigger self-censorship (Véliz, 2020; Büchi et al, 2022). Datafication risks turning the laboratory into a sort of panopticon, where individuals adjust their behaviour simply because they know that someone might be watching them (Foucault, 1977). This matters because the laboratory is usually a space that affords the individual a significant degree of autonomy, and this autonomy can be seen as important for the effectiveness and the creativity of the research process. The absence of in-depth surveillance can, for instance, give scientists the freedom to explore unorthodox protocols that could provide unexpected payoffs, but which the principal investigator (PI) might see as a waste of time. It is in these pockets of autonomy that they feel free and safe to try out new things and explore new routes. A system of deep dataveillance, or even just the possibility of such surveillance, can negatively affect how researchers, or experts more generally, work (Nguyen, 2022).

“Datafication risks turning the laboratory into a sort of panopticon, where individuals adjust their behaviour simply because they know that someone might be watching them.”

The issue of surveillance is not just about the relationship between employer and employee. It is also about the amount and type of information that third-parties gain, and whether they have the right to collect this information. For instance, does a company that sells microscopes or other research instruments have the right to know that a scientist was working on a Sunday afternoon? Do providers of ELNs have the right to know who the researcher communicates with?

Importantly, systems such as ELNs or LIMSs not only collect ‘activity data’ but might also be given licence to view and analyse the customer’s research data. This access is usually regulated through the “Terms of Service” with often broad justifications, such as “maintenance and improvement of services”. This effectively gives service providers a blank cheque to analyse the research activity of laboratories, providing them with a powerful real-time insight into what is happening where and when. Depending on the amount and variety of data that commercial providers of cloud-enabled tools can collect, they might have a better insight into the minute-by-minute workings of a research group than the head of the laboratory. It is not clear if researchers are always aware of the broad licence they grant to service providers, and if they would use these tools if they were aware of it.

## Does datafication change everyday research practice?

Another aspect of research that the datafication of science is likely to reshape goes right to the heart of the scientific process itself: the planning, execution and analysis of experiments. With the rise of cloud-enabled research we expect to see a shift in how and where crucial decisions are made. What materials or instruments to use; which sequence of steps to implement; or what analytic tools to apply to the data collected—most of these decisions will increasingly be made in the space of cloud-enabled systems, such as ELNs and LIMS. The architecture of these tools, their ways of enabling or blocking certain workflows, and the pre-installed tools or protocols they offer—all these elements will shape how scientists plan, perform and analyse experiments.

This influence matters, because these tools, like other sites of datafication, are not neutral; they are designed, in part at least, for better extraction of data about user preferences and behaviour (van Dijck, 2014). They are also designed to make commercial use of the insights they extract from the data. For instance, applications that are part of cloud-enabled research, such as ELNs and LIMS, may include systems that recommend specific reagents, kits or protocols to their users, tailored to their activity and preferences. Recommender systems, however, raise a variety of ethical challenges (Milano et al, 2020) and are likely to affect methodological choices, as they can function as persuasion tools (Gretzel and Fesenmaier, 2006; Burr et al, 2018).

It matters in this context that those who build the new systems and platforms are, more often than not, also the providers of reagents, instruments and analytic tools. The activity data that the new applications and systems collect thus have significant financial value for corporate players: understanding where, how, when and why consumables, instruments and services are used will allow them to better predict usage, and to recommend workflows that depend on specific products offered by the company and its commercial partners.

This is particularly relevant for contemporary science, which is often characterised by what Ulrich Krohs calls “convenience experimentation”: more and more experiments are done simply because they are convenient to do, thanks to the availability of ready-made kits or systems (Krohs, 2012). There is a real risk here that researchers will increasingly choose a certain method or use a kit not because it is best suited for the problem at hand, but because it is easy to use and integrate into (semi-)automated workflows.

“There is a real risk here that researchers will increasingly choose a certain method or use a kit not because it is best suited for the problem at hand, but because it is easy to use and integrate into (semi-)automated workflows.”

This is not to say that the commercial nature of some of these tools—there are not-for-profit options—is necessarily detrimental to the research process itself. The overall benefits might well outweigh potential downsides. But what is clear is that these tools are in a powerful position, as they sit at the heart of the experimental process. There is, then, a pressing need to develop a more in-depth understanding of the process of datafication and the tools that enable it, so that individual practitioners can better navigate the emerging datafied research landscape.

Part of the problem with developing such an understanding is that datafication is a complex and dynamic phenomenon. It will look different in different disciplines. And even within a discipline, different areas of research will encounter different issues over time. What I have described above, for instance, will mainly apply to laboratory-based sciences such as biology or chemistry. A field such as behavioural psychology might encounter different challenges and dynamics of datafication.

But even this limited analysis can already give some insights into how we might navigate the emerging datafied research environment. In the following I will propose three steps that can or should be taken when moving forward.

## Considerations for datafication

The first important step is rethinking the extent and nature of data management. Data management is already a central part of contemporary research. Researchers might talk about FAIR principles and Open Access infrastructures when they set up and manage their research projects. Funders want to see data management plans included in grant applications. And universities and corporations employ data officers who support and oversee data management practices.

But this focus on data management is usually limited to research data management (RDM). It is about the outputs of experiments, surveys and clinical trials, and not about the broader data that get collected in a datafied science. Once we shift our focus to datafication, we see that researchers, and the institutions at which they work, need to move towards a more integrated data management practice which also considers who collects data, what data is collected, what the data is being used for and how these wider data practices might affect the research process.

Part of such a more integrated data management practice entails a more sophisticated concept of datafication. It is tempting to reduce datafication to a focus on data, that is, to see it as an increase in the volume and variety of data that is collected. But whilst this is surely part of what is happening, more factors seem to be at play here. But what factors and why?

One answer to these questions can be found in a framework proposed by Anne Beaulieu and Sabina Leonelli in their book *Data and Society* (Beaulieu and Leonelli, 2021). They distinguish between four elements or “layers” of datafication: data, capacities, community and care. Beaulieu and Leonelli argue that, in order to understand the risks and the potential of datafication, we need to think about all these elements together. For instance, in different contexts there will be different communities that drive datafication, and these communities will value or care for data in different ways. How they care for and collect data will depend on the particular capacities that are available to them. Getting to grips with datafication thus means to think about all these elements at the same time.

Using such a more nuanced concept of datafication will not only provide a useful starting point for the development of integrated data management practices. It can also provide concrete guidance for researchers in the here-and-now. Take the example of a PI who needs to decide whether to switch the laboratory to an ELN, and if so to which one of the more than 70 ELN products that are available on the market (Kanza et al, 2017). If we think about datafication in a layered manner, the PI might consider in more detail the community that is involved in the service—is the product developed and run by fellow scientists, maybe as a non-profit or is it run by a commercial entity—and what capacities the service includes, such as is it “cloud native”, or can it be run on a local server. This can inform the choice the PI makes, depending on how they feel about the risk and potential of datafication. For instance, to protect their laboratory from excessive dataveillance, the PI might decide to use a product that can be run on a local server so they can define their own storage and data management protocols. This will come at a cost, as the laboratory will not be able to rely on services such as automated backups, software updates and maintenance. But it will provide them with more agency and privacy in their data practices.

This of course assumes that an individual PI can make their own decisions about what systems and practices to implement in their laboratory. This will not always be the case. Providers of commercial products will be keen to sell general licences to whole institutions, which will force, or at least encourage, all laboratories to use the same service. This will allow the seller to quickly grow their pool of users and hence data. For the institution, it will usually mean better interoperability between labs and potentially lower costs. But it also means that agency is taken away from individual researchers and PIs.

“Providers of commercial products will be keen to sell general licences to whole institutions, which will force, or at least encourage, all laboratories to use the same service.”

## The way forward

There will be no one-size-fits-all solution to the issue of datafication in research; whether the benefits outweigh the downsides will depend on a case-by-case analysis. What matters most is not having “the” answer but that the scientific community is aware of the phenomenon and that it has a critical discussion about what is happening and why. To enable these critical discussions there needs to be more empirical research on the phenomenon: what new tools and capacities are being brought into the laboratory and other sites of research, and how do they change the research process? And it will also be important to ensure that researchers have enough agency to make the decisions they think are best for their research or research group.

“What matters most is not having “the” answer, but that the scientific community is aware of the phenomenon and that it has a critical discussion about what is happening and why.”

The shift to cloud-enabled tools that will drive a deeper datafication of science is an exciting development with potentially great benefits for Open Science in terms of transparency, efficiency and reproducibility. But we also need to make sure that this shift happens in an informed and critically assessed manner.

### Supplementary information

Peer Review File
