# Supplementary material for: Surveillance in the lab? How datafication is changing the research landscape
Source: EMBO Rep. 2024 May 10;25(6):2525–8. doi: 10.1038/s44319-024-00153-2 (PMC11169247; doi:10.1038/s44319-024-00153-2)
Supplement: Supplementary file 1 — Peer Review File [file 44319_2024_153_MOESM1_ESM.pdf]

EMBO reports

Surveillance in the lab?

Stephan Guttinger

Corresponding author(s): Stephan Guttinger (s.m.guttinger@exeter.ac.uk)

Review Timeline:

Submission Date:	8th Mar 24
Editorial Decision:	22nd Mar 24
Revision Received:	17th Apr 24
Accepted:	26th Apr 24

Editor: Holger Breithaupt

Transaction Report: This article was editorially reviewed.
